# Moderna COVID-19 Vaccine: A New Player in Vaccine-Induced Thrombosis Without Thrombocytopenia

**DOI:** 10.7759/cureus.34015

**Published:** 2023-01-20

**Authors:** Harmehar K Kohli, Ankita Gore, Imran Baig, Viraj Modi

**Affiliations:** 1 Internal Medicine, Stony Brook University Medical School, Stony Brook, USA; 2 Internal Medicine, Stony Brook Hospital, Stony Brook, USA; 3 Internal Medicine, Northport Veterans Administration Medical Center, Northport, USA

**Keywords:** covid-19 vaccine side effects, covid-19, vaccine-induced thrombosis, hypercoagulability, covid-19 moderna vaccine

## Abstract

This is a case of a 31-year-old male with no past medical history who developed extensive pulmonary embolism (PE) and deep venous thrombosis (DVT) three days after receiving the second dose of the Moderna vaccine. The patient presented with left calf swelling and mild shortness of breath, with no fever or hemodynamic instability. Doppler ultrasound of the left lower extremity showed thrombus in the common femoral, superior, mid-, and distal femoral, popliteal, and posterior tibial veins. Chest CT angiography (CTA) visualized extensive pulmonary emboli in the bilateral main pulmonary, lobar, and segmental arteries.

Comprehensive hypercoagulable workup was unrevealing. The leading diagnosis was postulated as vaccine-induced thrombosis (VIT). Due to the diagnosis of bilateral sub-massive PE, the patient was initiated on enoxaparin and continued on direct-acting oral anticoagulation for at least one year.

Our report showcases a plausible link between the Moderna vaccine and thrombosis due to the extensive and unprovoked nature of DVT/PE in this patient with a negative hypercoagulable workup. Although the mechanisms behind the messenger ribonucleic acid (mRNA) and double-stranded deoxyribonucleic acid (dsDNA) vaccines vary, the possibility of vaccine-induced thrombosis (VIT) after the Moderna vaccine is highly probable. Vaccine-induced thrombosis should be considered in a patient presenting with unprovoked thrombosis after the Moderna COVID-19 vaccine, and further research needs to be conducted to ascertain the correlation. However, these findings should not dissuade the use of the Moderna vaccine given its proven benefits.

## Introduction

The significant effect of the COVID-19 pandemic prompted a collaborative global effort to develop effective and immediate vaccines against the virus. The messenger ribonucleic acid (mRNA)-1273 vaccine, also known as the Moderna vaccine, was available to the US public beginning in December 2020 and has since been administered around the world to millions of people. Data regarding the potential adverse effects of the vaccine continue to be collected as vaccines are administered, but the majority of vaccinations have been uncomplicated with the most common side effects being headache, fever, and fatigue [1]. Here, we present the case of a 31-year-old male patient who developed acute extensive pulmonary emboli (PE) and deep venous thrombosis (DVT) seven days after the administration of the second dose of the Moderna vaccine. This case report adds to a growing list of extensive DVT/PE being reported post-Moderna vaccine.

This article was previously presented as a meeting abstract at the Asian Pacific American Medical Student Association (APAMSA) National Conference in Columbus, Ohio, on January 8, 2022.

## Case presentation

The patient was a 31-year-old male with a past medical history significant only for obesity grade I (body mass index (BMI): 33 kg/m²) who presented to our emergency department (ED) with a four-day history of left leg swelling and pain in his left knee upon flexion.

He had received the second dose of his Moderna COVID-19 vaccine one week prior to presentation. The day after vaccine administration, the patient experienced fatigue, shortness of breath, sore throat, and nausea, which was self-limiting and resolved quickly. Three days after receiving the vaccine, the patient developed sharp and severe pain in the back of his left knee radiating down the back of his calf, with concomitant calf swelling. The pain increased upon exertion, prompting him to present to the ED. He took no daily medications except for a multivitamin and denied any recent travel, trauma, illness, chest pain, shortness of breath, excessive alcohol or drug use, or family history of blood clots or miscarriages.

**Figure 1:**
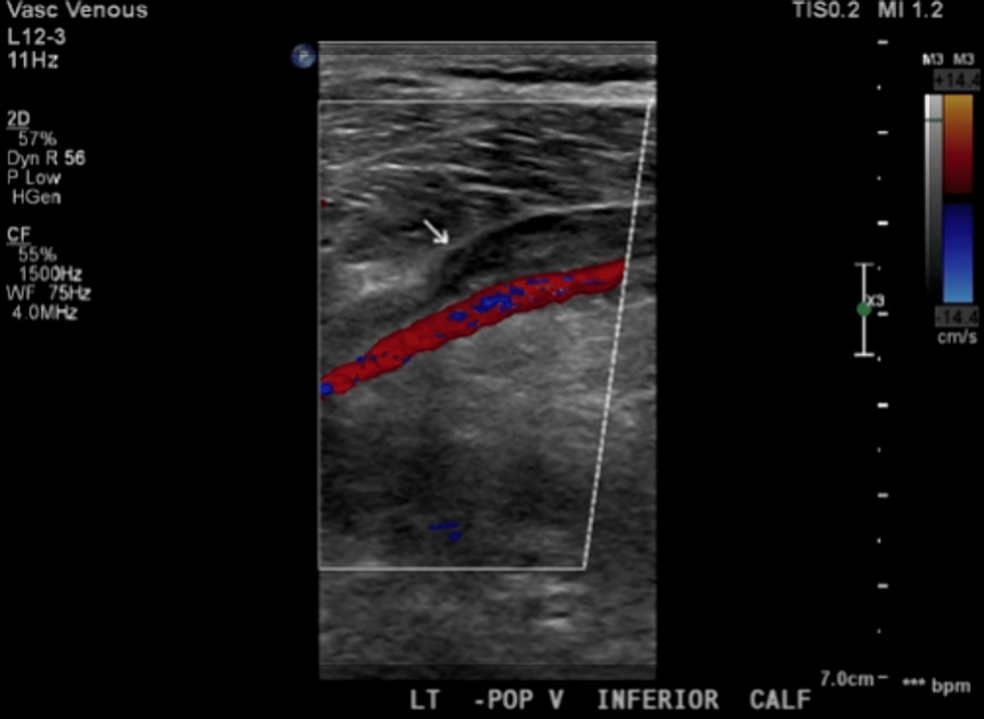
Doppler Ultrasound of the Left Lower Extremity

**Figure 2:**
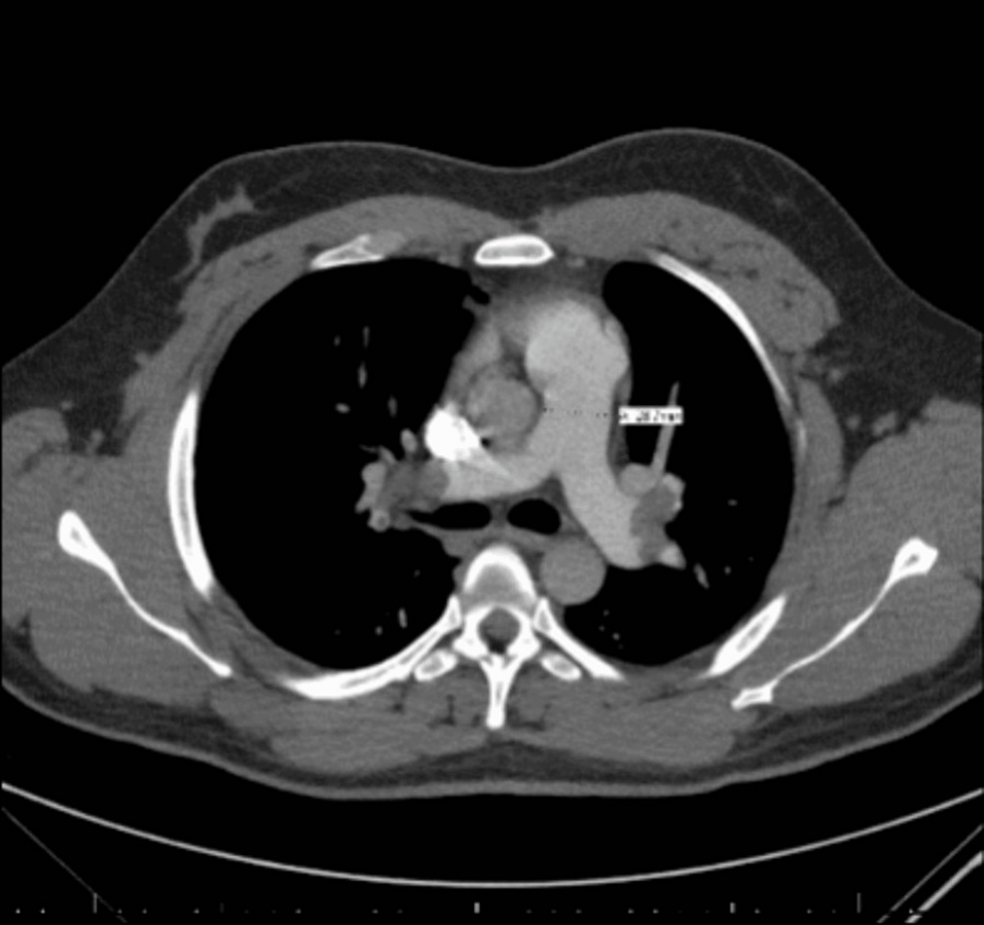
Chest CTA Showing Multiple Pulmonary Emboli CTA: CT angiography

In the ED, the patient had unremarkable vitals with a temperature of 97.7°F, blood pressure of 120/80 mmHg, respiratory rate of 18 breaths/minute, and heart rate of 78 beats/minute. On laboratory analysis, he had a D-dimer of 5,335 ng/mL D-dimer units (DDU), a negative troponin and normal lactic acid level, and otherwise unremarkable cell counts, electrolytes, and platelets (147 × 10^9^/L). Electrocardiogram (EKG) showed a prolonged QT interval and inverted T waves. Although he had no tenderness upon palpation of the left knee, a Doppler ultrasound of the left lower extremities showed a thrombus in the common femoral, superior, mid-, and distal femoral, popliteal, and likely posterior tibial veins (Figure 1). Chest CT angiography (CTA) found extensive pulmonary emboli in the bilateral main pulmonary, lobar, and segmental arteries with possible mild right heart strain, later confirmed by an echocardiogram (Figure 2).

Further workup of the deep venous thrombosis and extensive pulmonary emboli included hypercoagulable studies and workup of malignancy. Limited cancer screening, as outlined in the National Institute of Health Care Excellence Guidelines, proved to be negative [2]. Hypercoagulability studies were found to be negative, including heparin-induced thrombocytopenia (HIT) enzyme-linked immunosorbent assay (ELISA) and serotonin-release assay (Table 1). Given the negative diagnostic workup for pertinent etiologies, the leading diagnosis established by the hematology consultants was vaccine-induced thrombosis without thrombocytopenia.

**Table 1:** Workup of Thrombophilia Ab: antibody, GPI: glycoprotein I, OD: optical density

Laboratory variable	Reference range (adults)	Value
Factor V Leiden		No mutation found
Antithrombin activity level (%)	75-135	116
Beta-2 glycoprotein I Ab, IgG (GPI IgG units)	0-20	<9
Beta-2 glycoprotein I Ab, IgM (GPI IgM units)	0-32	<9
Cardiolipin Ab IgA (APL U/mL)	0-11	<9
Cardiolipin Ab IgG (GPL U/mL)	0-14	<9
Cardiolipin Ab IgM (MPL U/mL)	0-12	<9
Lupus anticoagulant		Not detected
Protein S total (%)	60-150	120
Protein S free (%)	57-157	74
Protein C activity (%)	73-180	78
Heparin-induced platelet Ab (OD)	0.000-0.400	0.045
Serotonin release assay		Negative

The patient was started on therapeutic anticoagulation in the ED. He initially received enoxaparin 110 mg every 12 hours (Q12h), which was transitioned to apixaban 10 mg twice per day (BID) for seven days, followed by apixaban 5 mg daily for at least one year. The patient is being followed by the hematology service outpatient and will be reevaluated for further anticoagulation in the future.

## Discussion

Vaccine-induced thrombotic thrombocytopenia (VITT) has recently received widespread global attention with numerous case reports detailing thrombotic complications after the ChAdOx1 nCoV-19 (AstraZeneca) and Johnson & Johnson/Janssen vaccinations [3]. Patients in these case reports have been found to have significant thrombocytopenia along with increased levels of platelet-activating antibodies against platelet factor 4 (PF4) [4]. Thus, the leading mechanistic theory resembles that of autoimmune HIT due to the high prevalence of antiplatelet factor 4 (PF4) antibodies in patients who developed thrombosis post-vaccine [5]. Although the mechanisms behind the mRNA and double-stranded deoxyribonucleic acid (dsDNA) vaccines are different, the possibility of vaccine-induced thrombosis after the Moderna vaccine has also been reported.

No definitive correlation has been made yet with the mRNA-based vaccines (Moderna and Pfizer-BioNTech); however, post-vaccine cases of immune thrombocytopenia and bleeding without thrombosis have been documented [6]. A previous case has been reported in which a patient developed popliteal and peroneal DVT after their second dose of BNT162b2 (Pfizer-BioNTech) vaccine; however, this patient was found to be heterozygous for factor V Leiden mutation, an underlying hypercoagulability that may increase the risk of thrombosis and be a possible confounder [7]. Another case series presented three patients who reported venous thromboembolism (VTE) after the mRNA-1273 vaccine; however, one patient was taking raloxifene, and another was currently on oral contraceptives (OCPs). Patients taking raloxifene have been found to have a slightly elevated risk of VTE, and patients taking OCPs have a relative risk of venous thrombosis of 3.5 compared to nonusers [8]. The patient presented in this current case report had no history of previous hypercoagulability and was taking no medications at the time of diagnosis.

Although the majority of cases involving vaccine-induced thrombosis have been associated with thrombocytopenia, Bhan et al. reported a case of DVT in a young female three days after her second dose of the Moderna COVID-19 vaccine [9]. The similarity of these two patients and the absence of thrombocytopenia suggest the pathophysiology behind post-Moderna DVT that may be different from adenoviral vector COVID-19 vaccines such as ChAdOx1 nCov-19 and Ad26.COV2.S.

Although it is possible that this patient’s DVT/PE could be an idiopathic event, the timing of the VTE immediately post-vaccine suggests that there may be a possible correlation.

## Conclusions

As vaccination efforts increase and new vaccines continue to arrive, it is imperative to identify the incidence of post-COVID-19 vaccine thrombotic events. Vaccination history should be obtained from all patients presenting with VTE without predisposing factors. Furthermore, continued reporting of these possible adverse events after the administration of COVID-19 vaccines is imperative in safely and properly caring for patients.

This report does not confirm a causative link between the vaccine and thrombosis, but rather, we suggest a plausible correlation due to the extensive and unprovoked nature of DVT/PE in this patient. Through this, we aim to shed light on a possible rare side effect of the Moderna COVID-19 vaccine to prompt awareness and future research studies.
